# Role of gut microbiota in thalassemia: a review of therapeutic prospects

**DOI:** 10.3389/fphys.2025.1523448

**Published:** 2025-03-19

**Authors:** Guanjun Chen, Yulan Li, Shirui Wei, Xinyu Wang, Zheshu Kuang, Weiming Guo, Jianbin Qin, Tianjun Huang, Youlin Li, Chunjiang Zhu

**Affiliations:** ^1^ Affiliated Hospital of Guilin Medical University, Guilin, Guangxi, China; ^2^ Shandong Second Medical University, Weifang, Shandong, China; ^3^ Chenzhou Third People’s Hospital (Group), Chenzhou, Hunan, China

**Keywords:** thalassemia, gut microbiota, therapeutic prospects, research progress, probiotics

## Abstract

In recent years, the study of gut microbiota has gradually become a research hotspot in the field of medicine, as gut microbiota dysbiosis is closely related to various diseases. Thalassemia, as a hereditary hemoglobinopathy, has a complex pathophysiological mechanism, and traditional treatment methods show limited efficacy. With a deeper understanding of the gut microbiome, researchers have begun to focus on its role in the pathogenesis of thalassemia and its therapeutic effects. This article aims to review the role of gut microbiota in thalassemia and its potential therapeutic prospects, analyze the latest research findings, and explore the impact and mechanisms of gut microbiota on patients with thalassemia, with the goal of providing new ideas and directions for future research and clinical treatment of thalassemia.

## 1 Introduction

Thalassemia is a hereditary hemolytic anemia caused by mutations or deletions in globin genes, with α-thalassemia and β-thalassemia being the most common types (Pinto and Forni, 2020; Baird et al., 2022). Epidemiological data show that thalassemia is widely distributed globally, with approximately 1.67% of the world population being carriers of thalassemia genes, mainly concentrated in the Mediterranean region, the Middle East, South Asia, and Southeast Asia (Paiboonsukwong et al., 2022; Alwi and Syed-Hassan, 2022; Yuson and Naranjo, 2022). In Southeast Asia, such as Thailand and Cambodia, the carrier rate of thalassemia is relatively high, which is related to the region’s long history of malaria prevalence. Some studies suggest that this gene mutation may act as a partial resistance mechanism against malaria (Williams and Weatherall, 2012). In China, the incidence of thalassemia is mainly concentrated in the southern regions, especially in Guangxi and Guangdong, where the carrier rate of α-thalassemia is as high as 15.43% in certain areas of Guangxi, and the carrier rate of β-thalassemia is 5.02% (Chen et al., 2022; Wang WD. et al., 2023a). Severe thalassemia patients require regular blood transfusions to maintain life, which places a heavy burden on both patients and their families. Although modern medical technology can significantly improve patient prognosis, the high cost of treatment and disparities in regional healthcare resources make this disease a public health challenge in many countries (Shah et al., 2019).

As a pivotal component of the host-microbe symbiotic system, gut microbiota maintains host homeostasis through nutritional metabolism and immunomodulation (Sorboni et al., 2022; Chen et al., 2021). Emerging evidence highlights its metabolites (e.g., butyrate) and microbiota-host crosstalk in hematological disorders, demonstrating potential regulatory roles in thalassemia pathogenesis (Wang et al., 2023b). Iron overload secondary to chronic hemolysis and transfusion in thalassemia patients significantly alters gut microenvironment, promoting proliferation of opportunistic pathogens (e.g., *Bacteroides*) while suppressing butyrate-producing bacteria (e.g., *Lactobacillus*) (Wang et al., 2020). This dysbiosis exacerbates disease progression through three key mechanisms: (Pinto and Forni, 2020) Reduced SCFAs production decreases expression of intestinal epithelial tight junction proteins, inducing gut barrier leakage and systemic endotoxemia (Lin and Zhang, 2017; Gill et al., 2022; Baird et al., 2022). Dysregulated microbial metabolism aggravates iron chelation imbalance, promoting intestinal free iron absorption and oxidative stress to establish pro-inflammatory microenvironment (Liu et al., 2020a; Yan et al., 2023; Paiboonsukwong et al., 2022). Depletion of beneficial species (e.g., *Bifidobacterium*, *Lactobacillus*) may impair iron absorption homeostasis, worsening anemia (Liu et al., 2022).

Recent studies revealed significant differences in gut microbiota diversity and composition between mild thalassemia and healthy pregnant women, particularly at genus and species levels (Lun et al., 2021). β-thalassemia patients exhibited significantly reduced gut microbial diversity compared to healthy controls, inversely correlated with iron overload levels (Nonejuie et al., 2024). Gut microbiota alterations correlate with quality of life indices, where dysbiosis-induced malnutrition may exacerbate anemia severity and comorbidities (Sriwichaiin et al., 2022). Thus, gut microbiota homeostasis emerges as a critical regulatory nexus bridging genetic defects in thalassemia and secondary complications (e.g., inflammation, iron overload).

Investigation of gut microbiota alterations opens novel therapeutic avenues for thalassemia management, particularly through personalized dietary interventions, fecal microbiota transplantation (FMT), and targeted probiotic/prebiotic formulations. For instance, butyrate-producing species (e.g., *Faecalibacterium prausnitzii*) modulate host iron metabolism via short-chain fatty acid (SCFA) production. Butyrate serves dual functions as both primary energy substrate for colonic epithelium and regulator of iron homeostasis, upregulating intestinal iron transporters (e.g., ferroportin) to enhance cellular iron export and mitigate overload (Xiao et al., 2024). Mechanistically, *F. prausnitzii* activates Nrf2 pathway to counteract iron overload-induced oxidative stress (Xiao et al., 2024). Therapeutic supplementation with *F. prausnitzii* or its metabolites shows potential to restore microbial equilibrium and ameliorate iron dysregulation-associated pathologies (Gough et al., 2024).

Emerging evidence reveals gut microbiota’s immunomodulatory capacity, suggesting microbiota-targeted interventions may synergistically improve systemic inflammation and clinical outcomes in thalassemia (Wang et al., 2023c; Pickard et al., 2017; Gao et al., 2018). Elucidating gut microbiota-thalassemia interactions provides dual benefits: deciphering disease pathophysiology and informing next-generation therapeutic development. This review synthesizes current advances in gut microbiota-thalassemia research, providing novel perspectives for elucidating disease mechanisms and developing innovative therapeutic strategies.

## 2 Thalassemia

**FIGURE 1:**
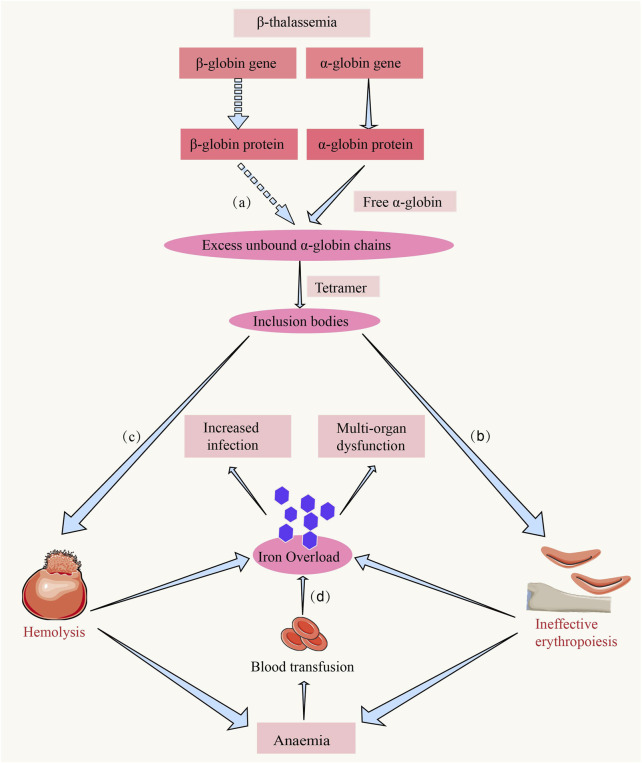
The hemolysis and ineffective erythropoiesis in β-thalassemia. **(a)** Due to the reduced or absent synthesis of the β-chain, the α-chain becomes relatively excessive. The excess α-chains cannot bind to β-chains, leading to the accumulation of free α-chains in erythroid precursors (Thein, 2018). **(b)** The accumulation of unpaired α-globin chains induces mitochondrial oxidative stress-mediated activation of the caspase-9/caspase-3 apoptotic cascade, triggering premature apoptosis of erythroid precursors at the proerythroblast stage and constituting the central pathogenic driver of ineffective erythropoiesis in β-thalassemia (Thein, 2018). **(c)** The imbalance of globin chains and deposition of α-chains in red blood cells lead to their rupture and clearance in peripheral circulation, a process referred to as hemolysis (Thein, 2018). **(d)** Iron overload is a common complication in patients with β-thalassemia, and the causes include chronic red blood cell transfusions, hemolysis, and ineffective erythropoiesis, leading to iron deposition and functional impairment in organs such as the heart and liver and the increased infection (Rachmilewitz and Giardina, 2011).

The genetic pattern of thalassemia follows Mendel’s first law of inheritance, with the globin genes located on chromosome 16 (α-chain) and chromosome 11 (β-chain). Different types of mutations result in reduced or absent synthesis of the α- or β-chain, leading to α-thalassemia or β-thalassemia, respectively (Jaing et al., 2021). Individuals carrying a single mutated gene are classified as thalassemia carriers and are generally asymptomatic; however, inheritance of two mutated genes results in mild to severe anemia (Diamantidis et al., 2023). The type and number of gene mutations determine the severity of the disease. In the case of β-thalassemia, mutations in the β-globin gene lead to reduced synthesis of the β-globin chain, resulting in abnormal red blood cell development, which further causes chronic hemolysis and ineffective erythropoiesis (Thein, 2018; Musallam et al., 2024) (Figure 1).

Currently, the primary treatments for thalassemia include regular blood transfusions and iron chelation therapy to prevent iron overload caused by transfusions (Rachmilewitz and Giardina, 2011; Muncie and Campbell, 2009). Hematopoietic stem cell transplantation is the only potential curative treatment for thalassemia; however, due to the limited availability of donors, only a small proportion of patients can undergo transplantation (Oikonomopoulou and Goussetis, 2021). In recent years, research on gene therapy and targeted drugs has advanced, offering new treatment options for thalassemia patients, especially lentivirus-based gene editing technologies, which have brought new hope for the treatment of β-thalassemia (Motta et al., 2020).

## 3 Gut microbiota

Gut microbiota refers to the microbial communities residing in the human digestive tract, including bacteria, viruses, fungi, and archaea (Chen and Wang, 2022). The human gut harbors over 1,000 different microbial species, with bacteria being predominant, primarily consisting of Bacillota, Bacteroidota, Actinomycetota, and Pseudomonadota (Bringer et al., 2022). These microorganisms maintain a complex symbiotic relationship with the host, participating in various physiological functions, including metabolism, immune regulation, and nutrient absorption (Adak and Khan, 2019). In a healthy gut, different bacterial species coexist and function in a dynamic balance (Lukáčová et al., 2023; Di Fusco et al., 2023). The diversity of a normal gut microbiota is considered key to its function, with higher diversity generally being associated with a lower risk of chronic diseases (Attaye et al., 2020). Gut microbiota helps the host digest indigestible dietary fibers, producing short-chain fatty acids (such as acetate, propionate, and butyrate), which provide energy to intestinal epithelial cells (Dalile et al., 2019). Additionally, gut microbiota synthesizes various vitamins (such as vitamin K and B vitamins) and participates in bile acid metabolism (Collins et al., 2023). These metabolic products not only help maintain the host’s energy balance but also play a crucial role in the integrity of the intestinal barrier and the host’s overall health (Winston and Theriot, 2020).

Gut dysbiosis refers to abnormal changes in the composition and function of the gut microbiota, usually characterized by a decrease in beneficial bacteria and an increase in harmful bacteria (Weiss and Hennet, 2017). When gut dysbiosis occurs, the host’s metabolism and immune system may be disrupted, potentially triggering various diseases. Studies have shown that gut dysbiosis is closely associated with obesity (Abenavoli et al., 2019), diabetes (Rodrigues et al., 2022), inflammatory bowel disease (Sugihara and Kamada, 2021), liver disease (Chopyk and Grakoui, 2020), and neurological disorders (Borrego-Ruiz and Borrego, 2024). In addition, abnormal changes in gut microbiota are also linked to the pathogenesis of various diseases, including cancer (Ahmad Kendong et al., 2021), allergic diseases (Niewiem and Grzybowska-Chlebowczyk, 2022), and autoimmune diseases (Miyauchi et al., 2023). Therefore, maintaining the balance of gut microbiota is crucial for the host’s metabolic health and immune stability, and modulating gut microbiota balance may offer new therapeutic approaches for various diseases (Dixit et al., 2021).

## 4 Gut microbiota in thalassemia research

### 4.1 Impact of iron metabolism disorders on gut microbiota composition

#### 4.1.1 The relationship between iron metabolism, iron overload, and gut microbiota

**FIGURE 2:**
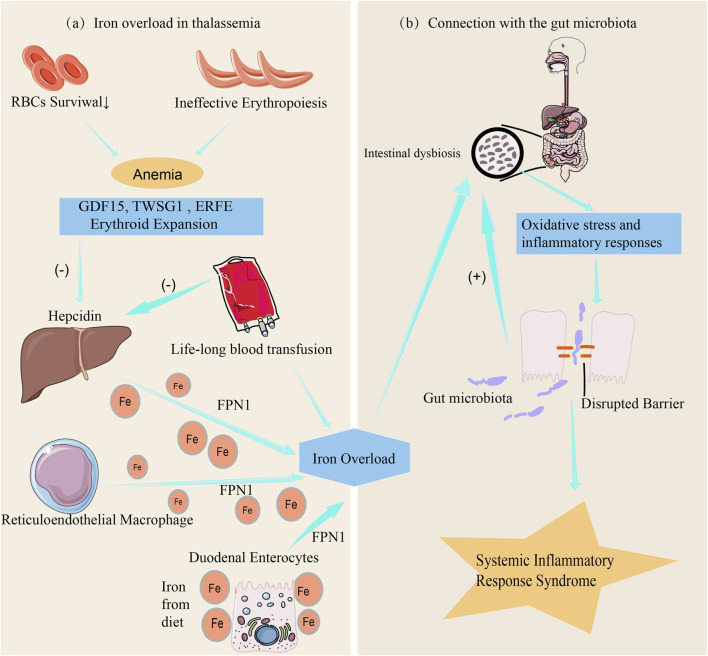
The link between iron metabolism, iron overload and gut microbiota. **(a)** Anemia resulting from ineffective erythropoiesis and shortened red blood cell (RBC) lifespan induces the production of erythropoietin, which in turn enhances erythropoiesis. The dramatic increase in erythroid expansion activates erythroid factors, including the secretion of GDF15, TWSG1, and ERFE. Excessive erythroid factors suppress the expression of hepcidin in hepatocytes, increasing iron absorption by duodenal enterocytes, while iron accumulation in hepatocytes and the reticuloendothelial system leads to iron overload (Saad et al., 2022). Additionally, chronic blood transfusions further suppress hepcidin expression in hepatocytes, exacerbating iron overload. **(b)** Iron overload affects the intestinal environment by triggering oxidative stress and inflammatory responses, leading to increased intestinal permeability, systemic inflammation, and exacerbation of gut microbiota dysbiosis (Luo et al., 2021).

There is a complex interaction between iron metabolism disorders and gut microbiota in patients with thalassemia. Under normal conditions, iron absorption in the gut is a finely regulated process, primarily controlled by factors such as hepcidin and transferrin receptors (Roemhild et al., 2021). However, patients with thalassemia (Figure 2a), especially those with severe forms, often rely on long-term blood transfusions to maintain red blood cell counts (Cazzola, 2022). One major issue associated with long-term blood transfusions is the significant increase in iron levels within the body, leading to excessive iron load, reduced hepcidin levels, increased iron storage, and elevated free iron concentration in the gut, resulting in iron overload (Chalmers and Shammo, 2016).

Iron is a critical element for the growth of many microorganisms. Iron overload increases the iron content in the gut environment, disrupting the balance of the gut microbiota, promoting the growth of pathogenic bacteria while inhibiting the proliferation of beneficial bacteria that rely on low-iron conditions, thereby altering the gut environment (Gu et al., 2023). Comparative analyses reveal distinct gut microbial dysbiosis in transfusion-dependent thalassemia (TDT) patients versus healthy controls, characterized by markedly reduced abundances of *Alistipes*, *Coprococcus*, and *Oscillospira* genera along with Ruminococcaceae family taxa. Conversely, *Clostridium* genus members (positively correlated with elevated ferritin levels) and bacterial taxa from Fusobacteriaceae, Enterobacteriaceae, and Peptostreptococcaceae families were strikingly enriched in patients. Crucially, α-diversity indices inversely correlated with serum ferritin concentrations, suggesting that iron overload may drive gut dysbiosis in TDT patients through redox-mediated microbial niche remodeling (Nonejuie et al., 2024). Furthermore, iron overload not only directly alters the iron concentration in the gut but also induces oxidative stress and inflammatory responses, further affecting the gut environment. It also impacts the expression of tight junction proteins, increasing intestinal permeability, which not only triggers systemic inflammatory responses but also exacerbates gut microbiota imbalance (Luo et al., 2021) (Figure 2b). It is the combined action of these factors that affects the diversity and function of the gut microbiota.

In summary, iron overload directly influences the iron content in the gut, disrupts the balance of the gut microbial community, and increases oxidative stress and inflammatory responses, significantly altering the gut microenvironment and thus affecting the diversity and function of gut microbiota. This dysbiosis of the gut microbiota further impacts the host’s metabolic and immune functions, exacerbating the symptoms and complications in patients with thalassemia.

#### 4.1.2 Potential therapeutic interventions

Modulating the balance of gut microbiota to properly manage iron overload may have a positive impact on the health management of thalassemia patients. For example, studies have found that *Cupriavidus metallidurans* can alleviate anemia by reducing iron deposition in the liver and spleen (Guo et al., 2024). This study is the first to improve anemia in mice by altering gut microbiota, proposing a novel strategy for the treatment of β-thalassemia.

Additionally, in the treatment of thalassemia patients, drugs directly targeting iron transporters (such as mini-hepcidin) can achieve similar iron transporter reduction effects, blocking hepcidin binding and preventing ferroportin internalization and degradation, thereby improving iron overload (Billesbølle et al., 2020; El-Beshlawy and El-Ghamrawy, 2019). Interestingly, Verma et al. found that the symbiotic bacterium *Bacteroides fragilis* can downregulate iron transporter expression and affect iron homeostasis in macrophages (Verma et al., 2019). Li et al. discovered that *Lacticaseibacillus casei* effectively improves iron-induced iron metabolism and gut microbiota dysbiosis, suggesting that effective treatment of iron metabolism may be related to changes in gut microbiota (Li and Liang, 2022). Therefore, gut microbiota may function similarly to iron transporter-targeting drugs by regulating iron homeostasis and improving iron overload, but further research is needed to determine whether this can be applied to thalassemia treatment. In summary, balancing iron metabolism and gut microbiota may become an important future direction for the treatment of thalassemia patients.

### 4.2 Gut barrier function damage and gut microbiota

Gut barrier function may be compromised by factors such as iron overload, oxidative stress, and chronic inflammation. These changes in the gut environment can further lead to gut microbiota imbalance, making the gut more susceptible to other complications and disruptions from the microbial community, increasing the risk of infections and inflammatory diseases in patients (Luo et al., 2021; Li et al., 2020; Di Tommaso et al., 2021). For example, oral iron administration in thalassemia mice induces more severe intestinal permeability (“leaky gut”), accompanied by an increase in fecal Gram-negative bacteria, leading to higher levels of endotoxemia and serum inflammatory cytokines (Visitchanakun et al., 2020).

Peerapat Visitchanakun et al. demonstrated that iron overload in thalassemic mice increased *Bacteroides* abundance while reducing populations of potentially beneficial Firmicutes taxa, including Lachnospiraceae and Clostridia (Visitchanakun et al., 2021). In β-thalassemia with iron overload, intestinal barrier dysfunction originates from multifaceted interactions among iron-induced oxidative stress, gut dysbiosis, and immune hyperactivation. Excess iron catalyzes reactive oxygen species (ROS) generation, disrupting tight junction proteins (e.g., ZO-1) and elevating intestinal permeability. Concurrently, iron enrichment promotes pathogenic Bacteroidales proliferation at the expense of beneficial Firmicutes, triggering endotoxemia via lipopolysaccharide (LPS) translocation. LPS activates the TLR4/NF-κB pathway in immune cells, driving pro-inflammatory cytokine (TNF-α, IL-6) release and neutrophil infiltration, which exacerbates mucosal damage through cyclooxygenase-2 (COX-2) upregulation. This inflammatory microenvironment perpetuates a vicious cycle of barrier deterioration and systemic bacteremia (Jandhyala et al., 2015). Probiotic interventions (e.g., *Lacticaseibacillus rhamnosus* GG) mitigate pathological damage by restoring microbial balance, enhancing short-chain fatty acid (SCFA) production, and suppressing TLR4/NF-κB signaling (Visitchanakun et al., 2021). Intriguingly, researchers observed that fecal microbiota transplantation (FMT) from iron-overloaded thalassemic mice to recipient mice over a 4-month duration—via co-housing with fecal gavage—failed to induce intestinal damage. This suggests that iron overload-induced gut dysbiosis contributes less significantly to mucosal injury in thalassemic mice compared to the direct toxicity of excess iron (Visitchanakun et al., 2021). However, conflicting evidence indicates that intestinal barrier disruption may alternatively arise from gut dysbiosis itself or pathogenetic taxa/metabolites associated with dysbiotic communities (Sriwichaiin et al., 2022). Therefore, more comprehensive and in-depth studies are needed to further investigate the complex relationship between gut barrier function damage and gut microbiota in thalassemia models.

### 4.3 The role of Th17 cells

Th17 cells are a type of helper T cell derived from CD4+ T cells that primarily secrete pro-inflammatory cytokines such as interleukin-17 (IL-17), playing a key role in mucosal immune defense, particularly in combating bacterial and fungal infections (Kanno et al., 2023; Yan et al., 2020). In recent years, Th17 cells have been regarded as an important component of the gut’s innate immune barrier. Their presence at appropriate levels helps maintain gut immune function, but excessive induction of Th17 cells can lead to gut dysfunction (Alexander et al., 2022).

#### 4.3.1 The relationship between gut microbiota and Th17 cells

The differentiation of Th17 cells is regulated by various signals, including metabolites produced by the gut microbiota (Alexander et al., 2022). Certain gut bacteria, such as Clostridia, *Lacticaseibacillus*, and *Bifidobacterium*, can produce metabolites like short-chain fatty acids (SCFAs), which promote Th17 cell differentiation by modulating the local immune microenvironment (Chen et al., 2019).

Additionally, gut microbiota can directly promote Th17 cell differentiation by inducing dendritic cells (DCs) and macrophages to secrete cytokines such as IL-6, IL-1β, and TGF-β (Wu and Wan, 2020). IL-6 and IL-1β are key pro-inflammatory cytokines that promote Th17 cell differentiation (Zhao et al., 2021), while TGF-β also aids in Th17 differentiation at certain concentrations (Wang et al., 2023d). The differentiation of Th17 cells relies on the coordinated interplay of multiple intracellular signaling pathways, with the JAK-STAT signaling pathway constituting one of the central regulatory axes. Cytokines IL-6 and IL-23 activate the JAK-STAT cascade upon binding to their cognate receptors, thereby inducing phosphorylation of STAT3. This post-translational modification is indispensable for both Th17 lineage commitment and functional activation, as STAT3 activation directly regulates the transcription of RORγt and IL-17A—hallmark molecular signatures of Th17 cells (Du et al., 2023). Therefore, gut microorganisms directly influence the generation of Th17 cells by regulating the expression of these cytokines.

**FIGURE 3:**
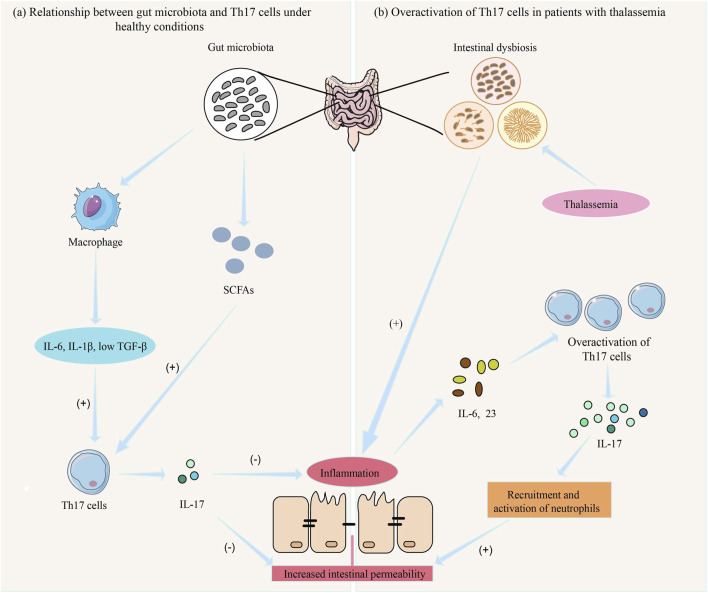
The role of gut microbiota and Th17 cells in inflammatory response in thalassemia. **(a)** Under normal conditions, the gut microbiota maintains intestinal immune function by producing SCFAs and activating macrophages to produce IL-6, IL-1β, and low levels of TGF-β, which induce Th-17 cell differentiation and the secretion of IL-17 (74, 83). **(b)** In patients with thalassemia, gut microbiota dysbiosis leads to intestinal inflammation, resulting in the release of IL-6 and IL-23, which causes excessive activation of Th-17 cells. This leads to the release of large amounts of IL-17, promoting the recruitment and activation of neutrophils, resulting in mucosal damage and increased intestinal permeability (Stein et al., 2018). Consequently, more pathogenic bacteria and toxins enter the body, triggering a systemic inflammatory response (Rendon and Choudhry, 2012).

As shown in Figure 3a, gut microbiota influences Th17 cell differentiation and function through various mechanisms, while Th17 cells regulate gut immune responses and mucosal barrier function, maintaining the balance between the host and gut microbiota (Liu et al., 2020b). However, when gut microbiota is dysregulated, excessive activation of Th17 cells may lead to chronic inflammation and gut damage (Zhou et al., 2023). For example, microbiota from patients with inflammatory bowel disease (IBD) increases the number of Th17 cells in the gut while reducing RORγt + Treg cells, exacerbating colitis in mice (Britton et al., 2019). Studies have shown that *Prevotella* primarily activates Toll-like receptor 2, leading antigen-presenting cells to produce Th17-polarizing cytokines and stimulating epithelial cells to produce IL-8, IL-6, and CCL20, promoting mucosal Th17 immune responses and neutrophil recruitment (Larsen, 2017).

#### 4.3.2 Overactivation of Th17 cells in thalassemia patients

Studies have shown that the proportion of Th17 cells in thalassemia patients is significantly higher than that in the normal population, and this increase is closely related to gut microbiota dysbiosis (Sun et al., 2023). Under healthy conditions, Th17 cells secrete IL-17, promoting intestinal epithelial cells to secrete mucus and antimicrobial peptides, thereby enhancing the defensive function of the intestinal barrier (Martínez-López et al., 2019). However, in thalassemia patients, long-term iron overload significantly alters the composition of the gut microbiota, leading to a reduction in beneficial bacteria, an increase in harmful bacteria, and an exacerbation of gut inflammation, especially due to the overgrowth of opportunistic pathogens (Nonejuie et al., 2024; Luo et al., 2021). This inflammatory environment in the gut further activates Th17 cells through a local pro-inflammatory cytokine network, such as IL-6 and IL-23 (84). The IL-17 and other factors released by Th17 cells not only enhance local immune responses but may also lead to mucosal damage and increased intestinal permeability by promoting neutrophil recruitment and activation. This allows more gut pathogens and toxins to enter the body, triggering a systemic inflammatory response (Rendon and Choudhry, 2012) (Figure 3b).

Additionally, this abnormal activation of Th17 cells may not be limited to the gut but could also affect the immune status of multiple organs through the circulatory system (Bayegi et al., 2023). Studies have shown that patients with severe β-thalassemia often suffer from liver, heart, and endocrine system damage due to iron overload (Karadag et al., 2020). This damage may be due to exacerbated inflammation triggered by immune responses (Baird et al., 2022), possibly involving cytokines produced by Th17 cells. Excessive IL-17 production by Th17 cells can enhance systemic inflammatory responses by promoting inflammatory reactions in endothelial and fibroblast cells (Barnes, 2016), which may contribute to the damage to the liver, heart, and endocrine systems in thalassemia.

#### 4.3.3 Potential therapeutic approaches to regulating Th17 cells and gut microbiota

Modulating gut microbiota to correct Th17/Treg imbalance has been shown to be a potential new method for treating myasthenia gravis (MG) (Chen and Tang, 2021), but whether this approach can be extrapolated to thalassemia treatment remains inconclusive. Given the key roles of gut microbiota and Th17 cells in the inflammatory response in thalassemia patients, therapies targeting gut microbiota and Th17 cells may provide new strategies for improving the prognosis of thalassemia patients. For example, studies have found that *F*. *prausnitzii*, *Bacteroides* spp., and *Roseburia intestinalis* may alleviate clinical symptoms of experimental colitis by regulating the Treg/Th17 cell balance and maintaining intestinal barrier integrity (Mohebali et al., 2023). Notably, bacterial intervention (particularly the three-strain consortium) significantly elevated splenic CD4+CD25+Foxp3+ Treg cell proportions, suppressed Th17 cell hyperactivation, and concomitantly reduced colonic IL-17A, IL-17F, and IL-22 levels while augmenting anti-inflammatory IL-10 expression. This treatment coordinately upregulated tight junction proteins Occludin and E-cadherin while downregulating pore-forming Claudin-2, collectively enhancing intestinal barrier integrity (Mohebali et al., 2023). Mechanistically, butyrate derived from *F*. *prausnitzii* inhibited HDAC1 activity, thereby stabilizing Foxp3 expression through histone H3K27 acetylation at its promoter region and disrupting IL-6/STAT3-mediated IL-17 transcription via STAT3 S727 phosphorylation blockade. This dual regulation maintained Th17/Treg balance and exerted potent anti-inflammatory effects (Zhou et al., 2018).

Probiotics have been shown to significantly modulate gut microbiota dysbiosis. In ulcerative colitis (UC), they can improve immune function and regulate inflammatory responses by modulating the Th17/Treg cell balance (Guo and Lv, 2023). For instance, the probiotic formulation VSL#3 exerts anti-inflammatory effects by generating conjugated linoleic acid (CLA) to activate PPARγ in macrophages, while concurrently suppressing NF-κB signaling to reduce macrophage infiltration (Isidro et al., 2014). *Bifidobacterium bifidum* FJSWX19M5 enhances intestinal barrier integrity through IL-10/IL-17 axis modulation and upregulation of tight junction protein expression (Qu et al., 2023). Additionally, *Lactobacillus acidophilus* attenuates Th17-mediated immune responses via STAT3 pathway inhibition and IL-23/IL-17 axis downregulation, accompanied by increased regulatory T cell (Treg) differentiation (Park et al., 2018). Inspired by this, restoring gut microbiota balance through probiotic supplementation and observing whether it helps reduce excessive Th17 cell activation and alleviates both gut and systemic inflammation in thalassemia patients could be a potential therapeutic direction.

In summary, gut microbiota dysbiosis and overactivation of Th17 cells in thalassemia patients may interact during disease progression, with both factors jointly exacerbating inflammation and immune disorders. Th17 cells may serve as an indirect indicator of thalassemia severity, and interventions targeting the Th17/Treg balance could represent new therapeutic targets for managing thalassemia and gut microbiota dysbiosis, as well as improving patient outcomes. Therefore, regulating the balance between gut microbiota and Th17 cell function could become a potential strategy for treating thalassemia patients in the future.

### 4.4 Exosomes and gut microbiota

Exosomes are extracellular vesicles released by various cells, carrying bioactive molecules such as proteins, nucleic acids, and lipids. They can transmit information between cells and regulate various biological processes, including immune responses, metabolic regulation, and cell growth (Yi and Kim, 2021). In thalassemia patients, long-term iron overload and chronic inflammation lead to significant gut microbiota dysbiosis. This dysbiosis not only affects local immune responses in the gut but may also contribute to systemic inflammation and metabolic abnormalities through exosomes.

#### 4.4.1 The regulatory role of exosomes between gut microbiota and the host

**FIGURE 4:**
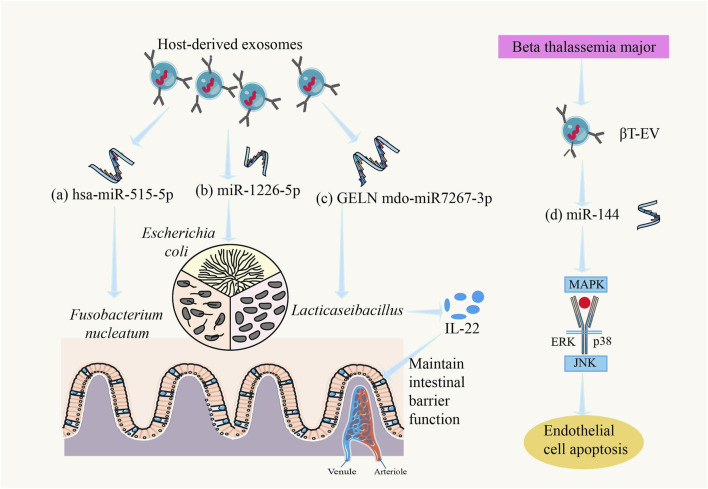
Regulatory role of exosomes in the gut microbiota and host. **(a)** hsa-miR-515-5p could promote the growth of *Fusobacterium nucleatum* (Liu et al., 2016), **(b)** miR-1226-5p could promote the growth of *Escherichia coli* (Liu et al., 2016), **(c)** GELN mdo-miR7267-3p could promote the growth of *LaActicaseibacillus*, causes the intestines to produce more IL-22 and maintain intestinal barrier function (Teng et al., 2018), and **(d)** βT-EV-induced endothelial cell apoptosis involved the MAPK/JNK signal-transduction pathway (Levin et al., 2021).

Recent studies have shown that there is a close relationship between exosomes (EVs) and gut microbiota. EVs play an important role as “messengers” between gut microbiota and the host, interacting with gut microorganisms to regulate the composition and function of the host’s gut microbiota (Yi and Kim, 2021). The effects of EVs on the gut include modulating gut microbiota, promoting intestinal cell proliferation, and alleviating intestinal inflammatory responses, with EVs from different sources having varied impacts on gut health (Ocansey et al., 2020) (Figure 4). For example, miRNAs in EVs can influence *Lacticaseibacillus* gene expression, promoting its proliferation and affecting its metabolism to produce more ligands for the aryl hydrocarbon receptor (AHR), which maintains intestinal barrier function. Activation of AHR can induce the production of more interleukin-22 (IL-22) in the gut, enhancing mucus secretion and preventing harmful bacteria from adhering to the intestinal epithelium (Teng et al., 2018). In alcohol-related liver disease (ALD), there is an interaction between exosomes and gut microbiota, which is considered one of the mechanisms underlying ALD pathogenesis (Cheng et al., 2024). EVs secreted by mesenchymal stem cells (MSCs) have been shown to effectively modulate gut microbiota in animal models of inflammatory bowel disease (IBD), but the mechanisms involved and whether this modulation can alleviate the characteristic dysbiosis associated with IBD require further study (Qiao et al., 2024).

MicroRNAs (miRNAs) are abundantly present in murine and human fecal samples, predominantly encapsulated within extracellular vesicles. Cell-specific ablation of the miRNA-processing enzyme Dicer demonstrated that intestinal epithelial cells (IECs) and Hopx-positive cells constitute the principal cellular sources of fecal miRNAs. These host-derived miRNAs exhibit cross-kingdom functionality, as evidenced by their internalization into commensal bacteria including *Fusobacterium nucleatum* and *Escherichia coli*, where they specifically modulate bacterial gene transcripts (e.g., *fadA* in *F. nucleatum* and *csgD* in *E. coli*) through sequence complementarity, thereby exerting dose-dependent regulatory effects on bacterial growth kinetics (Liu et al., 2016).

Additionally, EVs derived from the gut microbiota may be key mediators of microbiota-host communication. EVs from gut microbiota and probiotics can encapsulate various bioactive molecules, regulating important biological functions and exerting systemic effects on host health (Díez-Sainz et al., 2022). For instance, gut microbiota produce metabolites such as short-chain fatty acids (SCFAs) through the fermentation of dietary residues. These compounds not only provide energy to intestinal epithelial cells but are also transported to host cells via exosomes, modulating host metabolic and immune responses (Qiu et al., 2024). Furthermore, gut microbial communities influence neuronal growth, maturation, and synaptic plasticity through exosome-mediated signaling, suggesting their potential roles in neurodevelopment and neurodegenerative disorders (Kim et al., 2024). As mediators of intercellular communication, exosomes carry microbial-derived genetic material and metabolites that regulate tumor cell behavior, positioning them as novel therapeutic targets in cancer treatment (Shetty and Shetty, 2024).

#### 4.4.2 The role of exosomes in gut microbiota dysbiosis in thalassemia patients

In recent years, research on the relationship between thalassemia and exosomes has gained increasing attention. For example, in severe β-thalassemia (βT), EVs carry miRNAs to target cells, where βT-EVs induce endothelial cell apoptosis via the MAPK/JNK signaling pathway, and splenectomized βT-EVs induce bone marrow mesenchymal stem cell (BM-MSC) proliferation (Levin et al., 2021) (Figure 4). Additionally, studies have shown that exosomes can affect the expression of tight junction proteins in intestinal epithelial cells, altering intestinal permeability (Park et al., 2022). In thalassemia patients, this effect of exosomes may further worsen gut microbiota dysbiosis, exacerbate increased intestinal permeability, and allow more bacterial metabolites and toxins to enter the bloodstream, triggering systemic inflammation and complications.

Moreover, thalassemia patients often experience chronic inflammation, and exosomes may play a role in modulating the host’s inflammatory response by delivering inflammation-related cytokines, such as IL-6 and TNF-α (Arabpour et al., 2021). The transfer of these exosomes within the gut may aggravate the inflammatory state of the intestinal mucosa, further disrupting the balance of gut microbiota.

Due to long-term blood transfusions and iron chelation therapy, thalassemia patients experience iron metabolism disorders. Iron overload not only directly affects the composition of gut microbiota but also transmits information about abnormal iron metabolism in the gut to distant organs through exosome mediation, impacting multiple organs systemically. For example, one study investigated the effects of EVs from β-thalassemia patients with iron overload (+IO) and without iron overload (-IO) on cardiac cells, finding that EVs from patients with higher serum ferritin levels also contained higher levels of ferritin. This suggests that EVs from β-thalassemia patients carry iron-loaded proteins that induce cardiac cell proliferation (Atipimonpat et al., 2021). EVs are extensively involved in the complex processes of iron metabolism, carrying ferritin and expressing transferrin receptors. Bone marrow-derived EVs can even induce hepcidin expression in β-thalassemia. In turn, gut microbiota can influence iron homeostasis at the level of iron absorption and may affect macrophage iron recycling, but there is still no direct evidence that EVs interfere with this process (Daou et al., 2022).

#### 4.4.3 Exosomes as potential therapeutic targets

Given the critical role of exosomes in regulating gut microbiota and inflammatory responses, intervention strategies targeting exosomes could offer new therapeutic approaches to improving the health of thalassemia patients. Modulating exosome release or function could help restore gut microbiota balance, reduce inflammatory responses, and thereby improve iron overload and related complications.

### 4.5 lncRNA

Studies have demonstrated that long non-coding RNAs (lncRNAs) not only participate in transcriptional regulation but also play significant roles in various biological processes including intracellular signaling and cell cycle control. In thalassemia, specific lncRNAs have been found to correlate with clinical phenotypes in patients, potentially influencing disease severity through modulation of fetal hemoglobin (HbF) expression (Rujito et al., 2024). Notably, certain gut microbiota components may exacerbate iron overload impacts by regulating iron metabolism-related genes through lncRNA expression modulation (Sriwichaiin et al., 2022). Furthermore, microbial metabolites could affect cellular proliferation and apoptosis by altering the epigenetic status of host cells, thereby influencing lncRNA regulatory networks (Nonejuie et al., 2024). Our analysis of existing literature reveals that intestinal microbiota not only serves crucial functions in host metabolism, immune responses, and physiological homeostasis but may also regulate thalassemia-associated genes through lncRNA-mediated pathways.

#### 4.5.1 lncRNA H19

H19 is a widely studied lncRNA primarily associated with embryonic development and tumors (Rolla et al., 2021). Studies have shown that H19 overexpression increases miR-675 abundance, which inhibits the translation of mRNAs encoding tight junction protein ZO-1 and adherens junction protein E-cadherin, leading to epithelial barrier dysfunction (Zou et al., 2016). Research has confirmed that epithelial barrier dysfunction significantly affects the composition and function of gut microbiota, leading to gut microbiota dysbiosis (Cani and Everard, 2016). Meanwhile, in the hematological system of thalassemia patients, H19 expression levels may be regulated, affecting hemoglobin development and maturation. For example, Han Y et al. found that the H19/let-7/LIN28B axis may be involved in the γ-to-β-globin gene switch (Han et al., 2022). Therefore, H19 may influence disease progression by regulating hemoglobin synthesis, while also affecting the composition and function of gut microbiota by altering epithelial barrier function. Future research could further explore H19 as a potential target for regulating both thalassemia and gut microbiota balance.

#### 4.5.2 lncRNA MEG3

Studies have shown that MEG3 can inhibit inflammatory cytokines by regulating inflammation-related signaling pathways such as the NF-κB and p53 pathways (Li et al., 2022). The gut barrier is a dynamic system, and its disruption is often associated with inflammatory responses (Di Vincenzo et al., 2024). Overexpression of MEG3 may reduce intestinal inflammation, improve gut barrier function, and thereby influence the balance of gut microbiota. However, this hypothesis requires further and more extensive research for validation. In thalassemia, MEG3 may act as a hematopoietic regulatory factor, indirectly affecting the clinical manifestations of the disease. P53 activation, translational dysfunction, inflammation, imbalances in globin/heme synthesis, and autophagy dysregulation have been shown to disrupt erythropoiesis and impair red blood cell production (Liu and Karlsson, 2024). Given that thalassemia is characterized by ineffective erythropoiesis, MEG3 may modulate the p53 signaling pathway to affect the growth and differentiation of erythroid precursor cells, potentially alleviating or exacerbating anemia symptoms in thalassemia patients. Although direct studies on the link between MEG3, thalassemia, and gut microbiota are limited, MEG3 may play an important indirect role in the interaction between these factors by regulating systemic inflammation and erythroid cell growth and differentiation.

### 4.6 Potential regulation of hemoglobin synthesis by the microbiome

**FIGURE 5:**
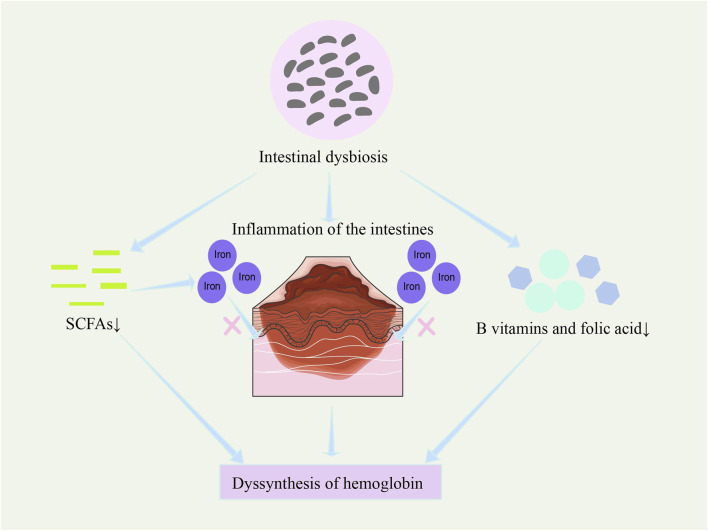
Potential regulation of hemoglobin synthesis by the microbiome. Gut microbiota dysbiosis affects hemoglobin synthesis by reducing iron absorption efficiency, increasing intestinal inflammation (Aksoyalp et al., 2023), decreasing the synthesis of B vitamins and folic acid (LeBlanc et al., 2013; Rubini et al., 2022; Koury and Ponka, 2004), and reducing the production of short-chain fatty acids (Klag et al., 2024).

In recent years, studies have found that the gut microbiome may play a role in regulating hemoglobin synthesis (Mahadea et al., 2021; Damian et al., 2021). In thalassemia patients in particular, gut microbiota dysbiosis appears to be linked to decreased hemoglobin synthesis capacity (Figure 5). Research indicates that gut microbiota dysbiosis may indirectly inhibit hemoglobin synthesis by reducing iron absorption efficiency and increasing intestinal inflammation (Aksoyalp et al., 2023). Intestinal inflammation disrupts gut barrier function and impairs normal iron absorption, while gut microbiota dysbiosis also alters metabolic pathways related to erythropoiesis and hemoglobin synthesis (Aksoyalp et al., 2023).

Gut microbes may affect hemoglobin synthesis not only by regulating iron metabolism but also through other metabolic pathways. For example, gut microbes can synthesize various vitamins (such as B vitamins and folate) (LeBlanc et al., 2013; Rubini et al., 2022), which are essential for the production and maturation of red blood cells (Koury and Ponka, 2004). Therefore, gut microbiota imbalance not only affects iron utilization but may also result in a deficiency of these essential nutrients, thus reducing the efficiency of hemoglobin synthesis.

Additionally, microbial metabolites, such as short-chain fatty acids, not only provide energy to intestinal epithelial cells and enhance iron absorption and transport but may also further regulate hemoglobin synthesis by influencing gene expression in host cells (Klag et al., 2024).

## 5 Gut microbiota modulation in thalassemia treatment

The gut microbiome plays a vital role in maintaining host metabolism, immune balance, and nutrient absorption (Ding et al., 2024). Thus, restoring the gut microbiome in thalassemia patients can not only help alleviate intestinal dysfunction but also improve overall health. Restoring gut microbiota can reestablish microbial balance by increasing the proportion of beneficial bacteria and reducing the overgrowth of harmful bacteria (Gagliardi et al., 2018), examples include probiotic supplementation, prebiotic administration, dietary interventions, and fecal microbiota transplantation (FMT). Additionally, restoring the microbiome helps enhance gut barrier function and reduce chronic inflammatory responses (Dixit et al., 2021).

### 5.1 Probiotics and prebiotics

Probiotics refer to live microorganisms that confer defined health benefits when administered in adequate amounts (≥10^9^ CFU/day) through mechanisms such as modulating the host’s gut microbiota or enhancing its functionality, with clinically validated specific strains (e.g., *Lacticaseibacillus rhamnosus* GG) serving as representative examples (Jäger et al., 2019).

The role of probiotics in enhancing nutrient absorption has been extensively investigated, particularly in thalassemia patients who frequently experience malabsorption due to disease characteristics and therapeutic interventions. Studies have demonstrated that probiotics improve nutrient assimilation through multiple mechanisms. For instance, they restore gut microbiota equilibrium and strengthen intestinal barrier function, thereby increasing nutrient bioavailability (Akter et al., 2020). Additionally, probiotics produce metabolites such as short-chain fatty acids (SCFAs), which support intestinal epithelial health and further optimize nutrient uptake (Judkins et al., 2020).

Chronic inflammation, a pathophysiological hallmark of thalassemia, is closely associated with disease progression. Probiotics exhibit anti-inflammatory potential via gut microbiota modulation, with mechanistic studies revealing their ability to regulate immune cell activity and secrete anti-inflammatory mediators (Ranganatha et al., 2024). Specifically, probiotics (e.g., *Lacticaseibacillus rhamnosus*) enhance regulatory T cell (Treg) differentiation and suppress pro-inflammatory cytokine production, collectively reducing systemic inflammation (Han et al., 2024). In ulcerative colitis (UC) patients, probiotic supplementation significantly decreases serum inflammatory markers, including C-reactive protein (CRP) and tumor necrosis factor-α (TNF-α) (You et al., 2024). These findings suggest that probiotics (e.g., *Lactobacillus acidophilus*, *Lactobacillus delbrueckii subsp. bulgaricus*, and *Bacillus subtilis*) not only improve intestinal health but also mitigate systemic inflammation to enhance overall patient outcomes. Furthermore, probiotics fortify the gut barrier by reducing intestinal permeability, thereby limiting bacterial endotoxin translocation into circulation—a critical benefit for immunocompromised thalassemia patients (Cai et al., 2025).

Prebiotics refer to non-digestible food ingredients that selectively stimulate the growth of beneficial bacteria, primarily consisting of dietary fibers and oligosaccharides (Gibson et al., 2017). Prebiotics promote the growth of beneficial bacteria, such as *Bifidobacterium* and *Lacticaseibacillus*, helping to restore gut microbiota diversity and stability, thereby improving metabolic and immune function in patients (Li et al., 2021). Research has demonstrated that specific prebiotics such as galactooligosaccharides (GOS) and fructooligosaccharides (FOS) enhance iron absorption by selectively stimulating the growth of beneficial gut bacteria. Mechanistically, prebiotics modulate gut microbiota composition, suppress pathogenic bacterial populations, and thereby improve iron bioavailability (Husmann et al., 2022). A randomized controlled trial in women revealed that combined administration of prebiotics with iron supplements significantly elevated serum iron levels and iron storage proteins (e.g., ferritin) (Apte et al., 2025). Animal studies further corroborate that prebiotic supplementation increases iron solubility, facilitating intestinal absorption (Iqbal et al., 2025). These findings suggest that prebiotics may offer novel therapeutic potential for alleviating iron overload in thalassemia patients.

Additionally, the immunomodulatory potential of prebiotics warrants particular attention. Studies indicate that prebiotics enhance host immune responses through gut microbiota modulation. For instance, protocatechuic acid (PCA)—a prebiotic derived from SV-53-fermented blueberry juice—significantly downregulates pro-inflammatory cytokines (e.g., IL-17A, IL-6, IL-23) to enhance immune tolerance and attenuate systemic inflammation (Shahbazi et al., 2023). In thalassemia patients, immune system integrity correlates closely with anemia severity. Prebiotic supplementation may improve immune function in these individuals, potentially alleviating anemia-related symptoms and improving quality of life (Donadio and Fabi, 2024). Notably, prebiotic dietary polysaccharides reduce intestinal permeability and reinforce barrier function, thereby mitigating bacterial translocation and endotoxin influx into systemic circulation—a critical benefit for thalassemia patients (Zhang et al., 2024). Collectively, prebiotics exhibit promising prospects for improving immune dysfunction in thalassemia.

Current preliminary clinical research has explored the therapeutic potential of probiotics and prebiotics in disease management. For instance, probiotic administration in neonates significantly reduces the incidence of necrotizing enterocolitis (NEC), offering novel strategies for clinical care in preterm infants (Khan et al., 2024). Prebiotics have been demonstrated to ameliorate hepatic fat content and metabolic parameters in patients with non-alcoholic fatty liver disease (NAFLD). A randomized controlled trial further revealed that polyphenol-rich prebiotics reduce hepatic fat deposition in overweight adults, thereby lowering the risk of NAFLD development (Agrinier et al., 2024). However, clinical trials targeting thalassemia patients are still limited, and further research is needed to validate the efficacy and safety of probiotics and prebiotics in this population. Theoretically, the application of probiotics and prebiotics can improve the gut microbiome in thalassemia patients, but their long-term effects and optimal dosages require further investigation.

### 5.2 Dietary intervention and gut microbiota modulation

Diet has a profound impact on gut microbiota, and appropriate dietary interventions can promote the growth and metabolism of beneficial bacteria (Beam et al., 2021), potentially improving the health of thalassemia patients. For example, the Mediterranean diet, rich in dietary fibers, prebiotics, and exogenous substances with antioxidant properties (such as vitamins C and E, selenium, and zinc), can promote the proliferation of beneficial gut bacteria, reduce inflammation, and improve gut health (Guasch-Ferré and Willett, 2021; García-Montero et al., 2021). Moreover, this diet emphasizes the consumption of fiber-rich vegetables, fruits, whole grains, olive oil, and fish while limiting red meat and processed foods, thereby reducing iron absorption and oxidative stress to mitigate the risks associated with iron overload (Awadallah, 2013; Scoditti et al., 2022).

### 5.3 Microbiota transplantation

Microbiota transplantation, especially FMT, involves restoring the gut microbial balance in patients by transplanting gut microbiota from healthy donors (Wang et al., 2019). In some studies, FMT has shown potential in restoring gut microbiota, reducing intestinal inflammation, and improving metabolic function (Antushevich, 2020). In thalassemia patients, FMT may help rapidly rebuild gut ecological balance, reducing complications caused by iron overload and intestinal inflammation.

The application of FMT has seen some success in treating other diseases, particularly in managing *Clostridioides difficile* infections and other gut-related disorders (Minkoff et al., 2023). However, research on FMT in thalassemia patients is still in its early stages. There are currently no large-scale clinical trials that definitively assess the effects of FMT on gut microbiota reconstruction and iron metabolism improvement in thalassemia patients, but this method has the potential to become an effective adjunctive therapy in the future.

### 5.4 Future applications

In the future, probiotics, prebiotics, and microbiota transplantation may become valuable adjuncts in the treatment of thalassemia patients. By modulating the composition of the gut microbiota and alleviating inflammation and oxidative stress caused by iron overload, there is potential to improve the overall health of patients (Apiwatsiri et al., 2022; Aabed et al., 2019; Anand et al., 2022). Probiotic and prebiotic therapies may be particularly suitable as long-term preventive interventions to maintain gut microbiota balance and prevent complications (Sikorska et al., 2023). FMT may serve as a rapid intervention for acute or severe dysbiosis, helping patients restore gut ecological balance (Cibulková et al., 2021).

**FIGURE 6:**
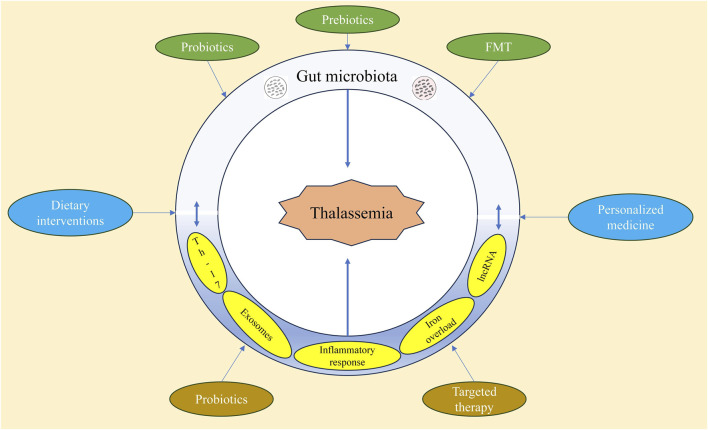
Regulation of intestinal microbiota in the treatment of thalassemia.

Combining probiotics, prebiotics, FMT, and dietary interventions to regulate the gut microbiota in thalassemia patients holds significant potential (Figure 6). Future clinical studies will further validate the effectiveness of these methods in improving the health of thalassemia patients.

As personalized medicine continues to advance, modulating the gut microbiota offers new possibilities for individualized treatment. Studies have shown that the composition and function of gut microbiota vary significantly among individuals, and these differences may influence drug metabolism and efficacy (Adak and Khan, 2019). For example, in the management of diabetes and obesity, specific gut microbiota can influence the host’s metabolic state by modulating short-chain fatty acid production, providing a basis for individualized treatment (Liu et al., 2021). Furthermore, personalized treatment can be tailored based on the patient’s genomic information and gut microbiota characteristics, enabling precise therapy that enhances efficacy and reduces side effects (Wullich et al., 2023). Future research could focus on developing personalized gut microbiota modulation strategies for thalassemia patients to explore its potential applications (Mack, 2024).

## 6 Conclusion

Gut microbiota has increasingly shown its importance in the study of thalassemia, with the interaction between the two occurring through mechanisms involving Th17 cells, exosomes, lncRNAs, and their connection to inflammation, immune responses, and disease progression.

Current studies indicate that the diversity and stability of gut microbiota are closely linked to the clinical symptoms of thalassemia patients, and alterations in gut microbiota may affect disease progression and patient quality of life. This discovery not only broadens our understanding of the pathological mechanisms of thalassemia but also provides new insights into potential intervention strategies.

As research into gut microbiota deepens, it emerges as a promising therapeutic target with unprecedented potential. Modulating the composition of gut microbiota through probiotics, prebiotics, fecal microbiota transplantation, or dietary interventions may improve overall health and alleviate symptoms in patients. Additionally, the concept of targeted therapies focusing on Th17 cells, exosomes, and lncRNAs opens new avenues for the treatment and management of thalassemia, suggesting that beyond traditional therapies, we can integrate microbiological perspectives to optimize current treatment strategies.

However, despite the broad prospects for research on gut microbiota and thalassemia, many challenges remain. First, there is significant individual variability in gut microbiota, and establishing standardized assessment criteria to evaluate its specific effects on thalassemia remains an urgent issue. Second, the scale and number of existing clinical trials are insufficient, and more systematic research is needed to verify the efficacy and safety of gut microbiota interventions. Additionally, researchers need to delve deeper into the interaction mechanisms between gut microbiota and the host to better understand its role in thalassemia.

In summary, gut microbiota holds significant clinical importance in the study of thalassemia, and further exploration in this field will bring new hope to patients. We call for more clinical trials and fundamental research to drive the development of this emerging field, ultimately aiming to improve the quality of life for thalassemia patients.
